# Challenges of validated decision support solutions in bipolar disorder: from actigraphy-based relapse detection to improving patient outcomes

**DOI:** 10.1192/j.eurpsy.2026.10372

**Published:** 2026-07-31

**Authors:** E. Bakstein, J. Schneider, M. Kolenic

**Affiliations:** 1 National Institute of Mental Health, Klecany; 2 Cybernetics, Czech Technical University; 3 Third Faculty of Medicine, Charles University, Prague, Czech Republic

## Abstract

**Aims:**

Symptom stabilization and prevention of clinical episodes are among the primary goals of clinical treatment of bipolar disorder (BD). It is complicated, among others, by the high intra-individual and inter-individual variability in long-term clinical course development. While digital tools offer great potential for symptom monitoring and clinical decision-making, demonstrating their clinical effectiveness and cost-effectiveness in the long term poses multiple challenges that have rendered past studies largely unsuccessful. We illustrate the potential and challenges using results from two longitudinal cohort studies in BD patients.

**Methods:**

The purely observational trial Aktibipo included 369 BD patients who were monitored for 18+ months using weekly self-assessment (ASERT), continuous wrist-worn actigraphy, and clinical evaluation (MADRS, YMRS, hospitalizations). The subsequent trial VALM1, used the same actigraphy and self-assessment monitoring and additionally included a digital intervention app (Mindpax), delivering targeted feedback and psychoeducation based on actigraphy and clinical status development. This follow-up trial included 86 patients, following up from the Aktibipo, and a group of 57 newly enrolled patients, monitored for 6 to 12 months. Clinical status, quality of life (Q-les-Q), and disability (WHODAS) were evaluated at three-month intervals.

**Results:**

A machine-learning classifier, trained on the Aktibipo study, showed balanced accuracy of 78%/80% when predicting episodes of depression/mania on the VALM1 cohort, using self-assessments and actigraphy. When validating the clinical impact of the system in VALM1, the AB group, which had previously used a monitoring system, did not show improvement after the intervention. The newly enrolled group showed significant improvement in WHODAS both at 6 months (d=-0.40, p=0.004) and 12 months (d=-0.41, p=0.046). The primary outcomes: QlesQ at 12 months, clinical status (MADRS, YMRS) and hospitalization rates did not show any improvement.

**Conclusions:**

The actigraphy provides warning signs of relapse, which have predictive power - especially when combined with self-assessments. The resulting implemented digital intervention showed improvement in disability but not in quality of life: while the QlesQ is an overall measure that covers a wide range of factors, the WHODAS better reflects the patient’s current state. The solution benefited newly enrolled patients only. Validation of reduction in relapse rates will require a novel approach to clinical validation of long-term digital therapeutic tools.

**Disclosure of Interest:**

E. Bakstein Shareolder of: Mindpax s.r.o., Grant / Research support from: Supported by OP JAK, grant no. CZ.02.01.01/00/23_020/0008560 and Czech Ministry of Health NU23-04-00534, Employee of: Mindpax s.r.o., J. Schneider: None Declared, M. Kolenic: None Declared.

